# Fellowship and future career plans for orthopedic trainees: gender-based differences in influencing factors

**DOI:** 10.1016/j.heliyon.2022.e10597

**Published:** 2022-09-12

**Authors:** Abdulaziz Z. Alomar

**Affiliations:** Division of Arthroscopy & Sports Medicine, Department of Orthopaedic Surgery, College of Medicine, King Saud University, Riyadh, Saudi Arabia

**Keywords:** Career plan, Gender, Orthopedic trainee, Fellowship, Resident, Fellow

## Abstract

**Objective:**

Understanding gender-based preferences and factors influencing fellowship and subspecialty choice might help maximize gender diversity in orthopedic surgery. This study aims to identify the differences between male and female orthopedic trainees' future career plans. It also examines the factors and determinants that influence selection of fellowship specialties in Saudi Arabia.

**Methods:**

This cross-sectional multicenter study recruited orthopedic surgical trainees from multiple training centers. The survey was completed by 270 participants; 20 residents were excluded because they were unsure of their future career interests or preferred fellowships in general orthopedic practice. The participants were either postgraduate orthopedic residents or fellows who already enrolled in postgraduate residency and/or fellowship training program. A total of 201 (80% of 250 participants) were male and 49 (20%) were female.

**Results:**

The orthopedic subspecialities and fellowship preferences and their influencing factors varied considerably across genders. Pediatric orthopedics and hand and upper extremity were top sub-specialties preferences among women while arthroscopy and sports medicine, arthroplasty, and trauma were the top preferences among men. Women preferred to practice single subspeciality while men preferred to do multiple fellowships and practiced different orthopedic subspecialities. The expected income, private-sector job opportunities, and hospital needs were the most significant factors influencing subspecialty and future career preferences among men while personal interest and social and family commitments were the most influential factors for women.

**Conclusions:**

This study addresses the knowledge gap regarding gender-based subspecialty preferences and the factors influencing them. The results can inform strategy development for addressing women’s needs and interests in orthopedic surgery as well as the shortages of orthopedic surgeons in some subspecialties. Furthermore, these data may assist directors of training centers in analyzing expected future demands on fellowship training programs and addressing the training gap in all subspecialties and career counseling.

## 1 Introduction

Trainees usually choose to subspecialize in orthopedics after finishing their residency with more than 90% performing fellowship training [1, 2, 3]. Although the number of female orthopedic residents is increasing, their numbers still lag behind those of male residents, especially in orthopedic surgery [4, 5, 6, 7, 8]. Furthermore, women pursue careers in orthopedics less than men in any other surgical specialty [6], and orthopedic surgery specialty has the lowest numbers of women trainees and attending surgeons [9]. While no gender-based differences in performance during residency have been reported [10], female fellowship applicants in orthopedics seem to have a higher matching rate compared to men [5]. Thus, the impression that women are less suited to being orthopedic surgeons appears to be inaccurate. Understanding career choice preferences and factors that motivate female trainees can help implement strategies that would increase the number of female applicants and improve gender diversity in orthopedics.

Female surgeons face many challenges with regards to cultural, social, and family commitments. Furthermore, little is known about how sex-based differences such as physical build, pregnancy, child care, and other factors like retirement age, working hours/days per week, burnout, workload, on-call duties, affect fellowship preference and career choices between men and women. Commonly perceived reasons for why orthopedics is not suitable for women are the need for physical strength, inability to have an appropriate work-life balance, and lack of mentorship [11]. Gender-based differences in the number of fellowship applicants, their match rate, and specialty choices have been extensively studied in Western countries [1, 2, 12], but the literature regarding this concern in Arabic countries is limited. Mentoring trainees of both genders in orthopedic career choice is an important issue that needs to be addressed, to improve future workforce distribution in orthopedics.

This study aimed to determine gender’s effect on orthopedic trainees' fellowship selection preferences and career choices and the factors and motives influencing those choices. To the best of the author’s knowledge, this is the first study that focuses on the influence of gender-based motives in the selection of, subspecialties, types of fellowship, and career choices in the field of orthopedic surgery.

## 2 Materials and methods

This cross-sectional study recruited orthopedic trainees across Saudi Arabia. Orthopedic trainees who planned to engage or were already engaged in fellowship training were included. Thus, the participants were either postgraduate orthopedic residents or fellows who had completed their postgraduate residency and were planning to pursue further fellowship training. Orthopedic trainees from registered centers were invited to participate in an anonymous questionnaire survey conducted on 2021.

The survey comprised of 32 online anonymous questionnaire items related to the potential factors influencing fellowship selection and future career. The first section of the survey included items on demographics and preferred fellowship specialty choices. The second involved items on influencing factors that may have affected participants' choices. These influencing factors were categorized into four themes [3]: training-related, work-related, specialization-related, and social-related. A 5-point Likert-type scale was used to rate the responses with options ranging from *strongly agree* to *strongly disagree*. Informed consent was obtained from all participants, and the study was approved by the King Saud University Institutional Review Board (approval date: 18.07.2021/IRB No. 21/0589).

### 2.1 Statistical methods

SPSS, version 23 (SPSS Inc., Chicago, IL, USA) was used for data entry and statistical analysis. Means and standard deviations (SDs) for the continuous variables were compared using a Mann-Whitney U test. Percentages of categorical variables were compared using the chi-squared test or Fisher’s exact test. A post hoc analysis was performed using the adjusted residual values to interpret a deeper inference on factors influencing the association between two categorical variables. Survey questions were grouped by theme. A gender-based comparative analysis was carried out in terms of these themed categories. Furthermore, a comparative analysis was performed separately for each influencing factor. All analyzes were performed at a significance level of 0.05.

## 3 Results

### 3.1 Group characteristics

Among 498 (71 female and 427 male) postgraduate orthopedic residents and fellows registered at the Saudi Council for Health Specialties (SCFHS), 420 potential participants were reachable and invited to participate in the survey. A total of 270 participants submitted their responses (response rate = 64%). Among the 270 participants, 13 (5%) were unsure of their future career interest and whether they would complete their fellowship programs, and seven male residents (3%) mentioned a preference for future careers as general orthopedists. Since these 20 participants did not meet the selection criteria, they were excluded from the survey. Therefore, a total of 250 eligible participants were included in this study. Among these 250 participants, 201 (80%) were men and 49 (20%) women. Among the male trainees, 84% were postgraduate residents and 16% fellows, while among the female trainees, 96% were postgraduate residents and 4% fellows.

Among the 250 participants, a significant number of male trainees had decided on their fellowship subspecialty in the third year of their residency training (31% out of 201 male trainees). Many women chose their fellowship preference in their second year of residency training (34%) (p = 0.04). The fellowships that most participants associated with higher burnout were spinal surgery and trauma. More female than male participants believed that trauma was associated with higher levels of burnout (94% vs. 74%, respectively, p = 0.01). More women than men also believed that spinal surgery was associated with higher burnout (63% vs. 48%, respectively, p = 0.04). No significant differences were found between the proportion of male and female participants planning to do their fellowship training abroad (36% vs. 37%, respectively, p = 0.823). However, there was a significant difference between the proportion of male and female trainees who preferred to do their fellowship locally (20% vs. 29%, respectively, p = 0.02).

### 3.2 Fellowship choices by gender

**Figure 1:**
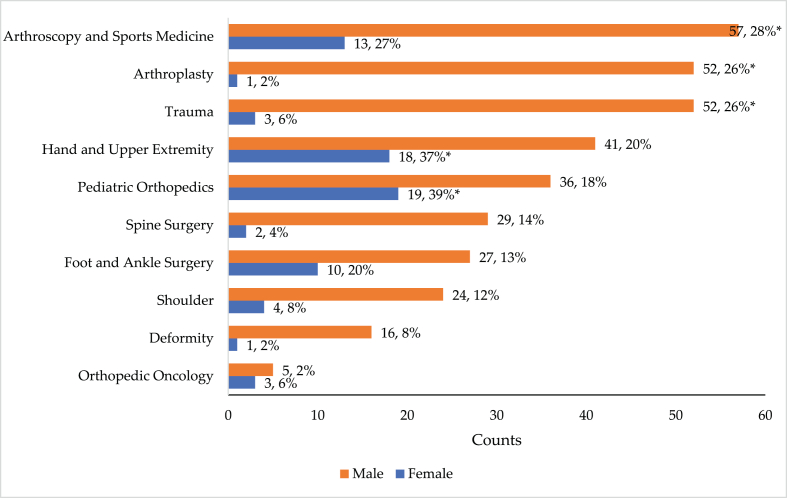
Fellowship choices for both male and female participants.

Significant differences were found in subspecialty choices; male participants were more likely to choose arthroscopy and sports medicine (n = 57, 28%), arthroplasty (n = 52, 26%), and trauma (n = 52, 26%), while female participants tended to choose pediatric orthopedics (n = 19, 39%) and hands and upper extremities (n = 18, 37%, p = 0.01) (Figure 1).

A significant difference between genders was found in terms of opting for single or multiple fellowships and subspecialties. 85% of male trainees preferred to pursue multiple fellowships and practiced more than one subspeciality, compared to 62% of female trainees (p-value = 0.001). Conversely, the percentage of women (38%) opting for a single fellowship and planning to practice one subspecialty differed significantly from men (15%), (p-value = 0.001).

### 3.3 Theme-based influencing factors

**Figure 2:**
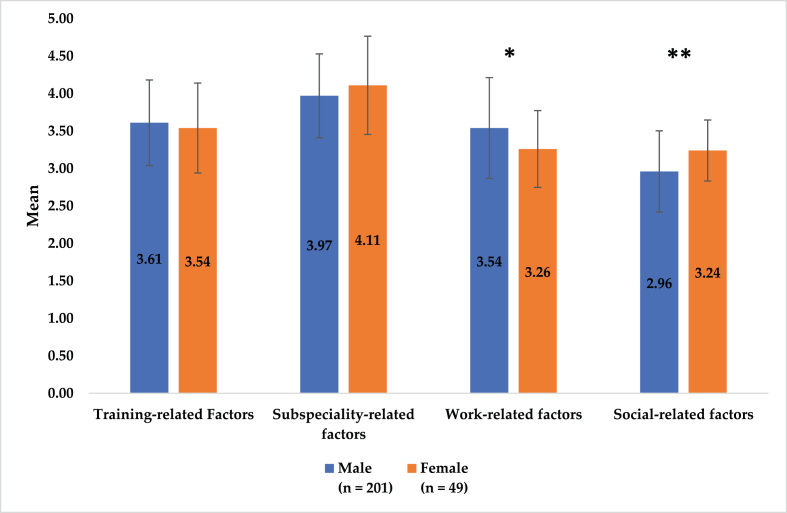
Overall female and male trainees' mean ratings for each themed-based influencing factors. ∗p = 0.006, ∗∗p = 0.001.

Significant difference between male and female participants was found for factors themed as work-related (mean [SD]: 3.54 [0.67] vs. 3.26 [0.51]) and social-related (mean [SD]: 2.96 [0.54] vs. 3.24 [0.41]) with p-values of 0.006 and 0.001, respectively. There was no significant gender-based differences in other theme-based categories (Figure 2).

### 3.4 Training-related influencing factors

The factor most perceived by both male and female trainees that influenced the fellowship and future subspeciality selection, was their experience during the residency training on the preferred subspeciality (mean [SD] = 3.93 [0.87] and 4.18 [0.73], respectively; Table 1). There were no significant differences between genders regarding training-related factors (Table 1).

**Table 1:** Comparison of influencing factors between male and female trainees.

Influential Factors	Male (n = 201) mean (±SD)	Female (n = 49) mean (±SD)	p-value
Training-related influencing factors			
Experience in your preferred subspecialty during residency training	3.93 (0.87)	4.18 (0.73)	0.079
Impact of life experience outside of medicine	3.41 (1.05)	3.18 (1.17)	0.283
To be more competent and strengthen an area of weakness	3.40 (1.03)	3.33 (1.23)	0.754
Impact of a mentor/role model you worked with during your residency	3.74 (1.01)	3.47 (0.98)	0.073
Subspeciality-related influencing factors			

Type of surgical skills practiced in the subspecialty	4.01 (0.81)	4.18 (0.76)	0.160
Variety of cases and patient volume	3.79 (0.88)	4.02 (0.78)	0.108
Type of patient population and disease pathology	3.86 (0.86)	4.02 (0.78)	0.560
Surgical intervention outcomes and disease prognosis	4.21 (0.75)	4.22 (0.90)	0.586
Work-related influencing factors			

Opinion, request, or advice from institution/department chair	2.99 (1.15)	2.98 (1.07)	0.869
Do your hospital needs affect your decision?	3.83 (1.06)	3.18 (1.05)	***0.001*∗**
Expected work/life balance, lifestyle, and call responsibilities	3.58 (1.03)	3.84 (0.90)	0.065
Expected income	3.74 (0.93)	3.00 (0.74)	***0.001*∗**
Job opportunities in the private sector	3.60 (1.03)	2.80 (0.91)	***0.001*∗**
Guarantee of jobs after finishing your fellowship	3.51 (1.23)	3.76 (1.11)	0.262
Social-related influencing factors			

Personal interest	4.15 (0.81)	4.57 (0.71)	***0.001*∗**
Family and social commitments	3.01 (0.94)	4.10 (0.65)	***0.001*∗**
Prestige of selected subspeciality in society	2.81 (1.02)	2.57 (0.84)	0.093
Friends and/or family advice	2.52 (1.01)	2.53 (0.94)	0.875
The fact that you are male or female	2.30 (1.06)	2.41 (1.29)	0.705

### 3.5 Subspecialty-related influencing factors

Surgical outcomes and disease prognosis were rated as the highest influencing factor by both male and female trainees (mean = 4.21 and 4.22, respectively; Table 1). However, there was no significant difference between men and women with regard to all subspeciality-related factors (Table 1).

### 3.6 Work-related influencing factors

Male trainees ranked the subspecialty requirements of hospitals located in the geographic areas where they planned to work as the most influential factor for their fellowship choice (mean [SD] = 3.83 [1.06]; Table 1). Female trainees considered work/life balance, workload, and call responsibilities to be the highest influencing factors (mean [SD] = 3.84 [090]; Table 1).

For work-related influencing factors, male trainees were significantly more influenced by hospital needs (mean: 3.83 vs. 3.18, p-value = 0.001), income expectations (mean: 3.74 vs. 3.00, p-value = 0.001), and private-sector job opportunities (mean: 3.60 vs. 2.80, p-value = 0.001). On the other hand, there were no significant differences between men and women in their opinions regarding other work-related influencing factors.

### 3.7 Social-related influencing factors

When examining the social factors influencing fellowship selection for both groups, more women were significantly influenced by personal interest (mean: 4.57 vs. 4.15, p-value = 0.001) and social and family commitments (mean: 4.10 vs. 3.01, p-value = 0.001). On the contrary, no significant difference was found between male and female trainees regarding other social-related influencing factors (Table 1).

## 4 Discussion

In Saudi Arabia, according to the SCFHS statistics for 2021 residents, women account for 14% of orthopedic residents. Despite being low, this percentage is comparable to the upper range in Western countries such as the United States, where women comprise 12–15% of the residents in orthopedic surgery [4, 5].

Though the literature addresses the reasons for why women are underrepresented minorities in orthopedic residency training and why women choose the orthopedic specialty, little is known about their preferences for fellowship and subspecialty. Understanding the subspecialty preferences of women in orthopedics and the factors influencing their choices could help change the relatively low proportion of women’s participation in orthopedics.

In this study, none of the female trainees surveyed were willing to practice as generalists, and all of them had plans to pursue training in at least one subspecialty. Contrarily, 3% of the male residents planned to practice as generalists. The current findings are consistent with those of Hariri et al., who found that preference for subspecialty rather than generalist practitioner was significantly more in women than in men (62% vs. 34%, respectively) [12].

Despite the fact that 71% of the women in this study were unmarried, female trainees significantly ranked social and family commitments as important factors influencing their decision for subspecialty, compared to men. This could be because their present decisions are based on future family plans. Women have social commitments and other priorities and responsibilities in life, such as pregnancy and child-raising, and these commitments could impact their work productivity [13]. Furthermore, it has been reported that female physicians in the primary care specialty have been found to provide fewer clinical services and retire five years earlier than male physicians [14]. Hariri et al. found that significantly more women than men planned to change to part-time status or reduce their work hours at some time during their careers [12]. Amoli et al. found that the women’s weekly workload and surgical case volume are lower than men’s, and 26% of men reported performing more than seven surgeries per week compared to 10% of women [15]. Madhuri and Khan surveyed 221 female orthopedic surgeons and trainees in India and found that 40% of them found the work/life balance to be a challenge, and 60% felt that they were able to achieve their full working potential [16]. Nearly half reported that family life and planning were affected, and 75% felt a lack of maternity support. Although they all felt competent to do orthopedics, 11% wanted to leave the field [16].

Another important finding of this study was that female trainee preferred pediatric, hand, and upper extremity specialties while male trainees preferred sports medicine, arthroplasty, and trauma subspecialties. This is consistent with previous studies; Rohde et al. surveyed 232 female orthopedic members of the Ruth Jackson Orthopaedic Society and found that hand (24%), pediatric orthopedics (19%), and sports (15%) were the most common subspecialties chosen [11]. Hariri et al. reported that significantly more women planned to pursue a pediatric fellowship than men (24% vs. 6%, respectively), and significantly more men were planning to pursue a sports fellowship (31% vs. 11%, respectively) [12]. Cannada found that highest proportion of women (25%) were doing pediatric orthopedic fellowships, followed by foot and ankle (14%), and the spine had the lowest proportion (3%) [5]. The low percentage of women participation in major subspecialties like trauma and spine could be because women, more significantly than men, perceive burnout to be highly associated with these two subspecialties.

There are not many studies that have surveyed orthopedic trainees to investigate the factor influencing their fellowship subspeciality selection. Financial incentives such as increased income and increased opportunities to secure jobs in the private sector were found to be more influential for men than for women in this study. Previous studies have explored the influence of these factors on pursuing fellowship training in general, where they reported that economic gain was an important factor for undergoing fellowship training [17, 18]. Studies have also found that fellowship-trained orthopedic surgeons have increased job opportunities compared to non-fellowship-trained surgeons [17]. Furthermore, a fellowship in orthopedics can yield positive returns in some subspecialities [18]. Hariri S et al. have observed that orthopedic residents prioritized intellectual factors and role models/mentors for determining their fellowship specialty [19]. Similarly, a survey by Kavolus et al. found that intellectual stimulation and possible exposure to diverse cases influenced fellowship selection [20]. While the findings of this study support the results of previous studies on factors determining fellowship preferences, studies that compare male and female trainees' preferences are limited. Amoli et al. surveyed recent fellowship graduates of pediatric orthopedic and found that a higher percentage of men (compared to women) reported that finances were important when selecting a job [15]. Butler et al. recently investigated gender-based differences in factors influencing fellowship selection. They found that females were significantly influenced by academics, intellectual interests, and patient interactions while males considered earning potential to be more important [1]. Rohde et al. reported that the most common reasons women choose orthopedic surgery as a specialty were enjoyment of manual tasks, professional satisfaction, and intellectual stimulation [11].

Interestingly, there were no significant differences between men and women with regards to the influence of role models' or mentors' on trainees' choice of subspecialty. However, sub-analysis of fellowships frequently selected by men (sports medicine) and women (pediatric) showed that male trainees identified mentors/role models as an influential factor significantly more often than female trainees. Some studies have reported that one possible cause for the smaller number of female medical students choosing orthopedics is the lack of female mentors or role models among orthopedic surgeons and faculty in training programs [11, 21]. It has been reported that residency training programs with higher numbers of female residents had higher proportions of women in the faculty and leadership positions, suggesting better access to same-sex mentors for female trainees [22]. Prior studies have suggested that mentors were deemed influential in determining the subspecialty, enhancing performance, encouraging workforce engagement, and contributing to learning promotion [23, 24, 25]. Brook et al. found that female medical students may lack access to mentoring opportunities because mentoring experiences are less prevalent in medical schools [23]. Jurenovich and Cannada found that 79% of female trainees did not report mentorship as a motivating factor; this could be due to the low percentage of female surgeons in orthopedics compared to other surgical specialties [26]. The role of mentorship in subspecialty selection was agreed upon by both male and female trainees, although there was no significant difference between them.

The strength of this study is its discussion of factors that influence female participation in orthopedic fellowship programs, which has scarcely been addressed in the literature. The study relies on statistically significant comparisons to highlight important gender differences in the preference for subspeciality fellowship and the variations in the factors that influence their subspeciality selection (social factors for women and work-related factors for men). The study being multicentric provides insights on orthopedic trainees from different regions of Saudi Arabia. The survey being based on multiple themes i.e., training-related, work-related, specialty-related, and social-related factors, provides a comprehensive evaluation of orthopedic trainees' preferences in fellowship selection. The influential factors identified in this study can help policy makers customize fellowship opportunities, improve women's experience, and motivate them towards a better career in orthopedics.

There were some limitations to this study. First, it was cross-sectional and voluntary survey-based with potential for both sampling and response bias. Second, the factors influencing subspecialty choice could change during residency [1]. Although most trainees decide their career in the second and third postgraduate years, as their training progresses and their exposure to other orthopedic subspecialties increases, they may change their minds and eventually not undertake their original planned subspecialties. Third, it is difficult to comprehensively list all factors influencing the subspecialty decision, and some important factors may be missing. Lastly, the current study presents the preferences and its influencing factors for orthopedic trainees who are already enrolled in residency and/or fellowship training program, and their preferences may be different than those who have already finished their training and started practicing orthopedic independently. However, this aspect was not investigated in the current study.

## 5 Conclusion

This study addresses the gap in knowledge regarding gender-based subspecialty preferences and the factors influencing those preferences. The results can help decision makers and program directors design and implement strategies that address the needs and interests of women in orthopedic surgery and deficiencies of surgeons in some specific orthopedic subspecialties.

The data shared in this study provide a foundation that may assist stakeholders and policymakers in analyzing expected future demands on fellowship training programs. The results elucidate female orthopedic trainees' subspecialty preferences and their influential factors. This information can be a valuable resource for career counseling for women in orthopedics and for developing strategies to minimize gender disparities in the field.

## Declarations

### Author contribution statement

Abdulaziz Z Alomar: Conceived and designed the experiments, Performed the experiments, Analyzed and interpreted the data, Contributed reagents, materials, analysis tools or data, Wrote the paper.

### Funding statement

This research did not receive any specific grant from funding agencies in the public, commercial, or not-for-profit sectors.

### Data availability statement

Data will be made available on request.

### Declaration of interests statement

The authors declare no conflict of interest.

### Additional information

No additional information is available for this paper.
