# Corrigendum to “Fellowship and future career plans for orthopedic trainees: Gender-based differences in influencing factors” [Heliyon 8(9) (September 2022) e10597]

**DOI:** 10.1016/j.heliyon.2023.e16550

**Published:** 2023-05-29

**Authors:** Abdulaziz Z. Alomar

**Affiliations:** Division of Arthroscopy & Sports Medicine, Department of Orthopaedic Surgery, College of Medicine, King Saud University, Riyadh, Saudi Arabia

In the original published version of this article, the corresponding author email (dr_abdulaziz@yahoo.com) has been hacked multiple times and unfortunately, they won't be able to use it regularly in future. This email has been changed to:

“azalomar@ksu.edu.sa”

The authors apologize for the errors. Both the HTML and PDF versions of the article have been updated to correct the errors.

